# Prevalence and correlates of skin examination among ethnically diverse young adult survivors of childhood cancer

**DOI:** 10.1002/cam4.5520

**Published:** 2022-12-09

**Authors:** Kimberly A. Miller, Angela A. Li, Katherine Y. Wojcik, Julia Stal, Myles G. Cockburn, Gino K. In, David R. Freyer, Ann S. Hamilton, Joel E. Milam

**Affiliations:** ^1^ Department of Population and Public Health Sciences Keck School of Medicine of the University of Southern California Los Angeles California USA; ^2^ Department of Dermatology Keck School of Medicine of the University of Southern California Los Angeles California USA; ^3^ Spatial Sciences Institute, Dana and David Dornsife College of Letters, Arts and Sciences University of Southern California Los Angeles California USA; ^4^ Department of Medicine Keck School of Medicine of the University of Southern California Los Angeles California USA; ^5^ USC Norris Comprehensive Cancer Center Los Angeles California USA; ^6^ Cancer and Blood Disease Institute, Children's Hospital Los Angeles Los Angeles California USA; ^7^ Department of Pediatrics Keck School of Medicine of the University of Southern California Los Angeles California USA; ^8^ Department of Epidemiology and Biostatistics, Program in Public Health, Chao Family Comprehensive Cancer Center University of California Irvine California USA

**Keywords:** follow‐up care, secondary prevention, skin cancer, survivors of childhood cancer

## Abstract

**Background:**

Skin cancer is the most common secondary malignancy among young adult childhood cancer survivors (YA‐CCS). Skin examination to detect skin cancer early (including melanoma as well as basal or squamous cell skin cancers), both physician‐based (PSE) and self‐skin exam (SSE), is recommended, particularly for radiotherapy‐exposed YA‐CCS who are at high risk of developing skin cancer.

**Methods:**

Awareness and prevalence of skin examination and demographic, clinical, and healthcare correlates were examined in a population‐based sample of YA‐CCS with diverse cancer types excluding melanoma. Descriptive frequencies and logistic regression models were conducted using sample weights to correct for non‐response bias with PSE, SSE and adherence to both as outcomes.

**Results:**

The sample comprised 1064 participants with 53% Latino. Eight percent of participants were aware of the need for skin examination; 9% reported receipt of PSE within past 2 years; 35% reported regular SSE; and 6% were adherent to both. Among the radiotherapy‐treated, 10% were aware of the need for skin examination, 10% reported recent PSE; 38% reported regular SSE; and 8% were adherent to both. Healthcare and clinical factors including healthcare self‐efficacy, engagement in cancer‐related follow‐up care, greater treatment intensity and greater number of treatment‐related late effects were positively associated with PSE and SSE. Latino YA‐CCS were less likely to engage in PSE and SSE.

**Conclusion(s):**

Adherence to recommended screening for skin cancer was low in this at‐risk population, notably for YA‐CCS exposed to radiotherapy. The development of effective strategies to expand skin cancer screening is needed in this at‐risk population.

## Graphical abstract

Despite the high risk of skin cancer as a second malignancy among young adult survivors of childhood cancer, in this study extremely low rates of skin screening (both physician‐based and skin self‐examination) were found in a diverse population‐based sample.

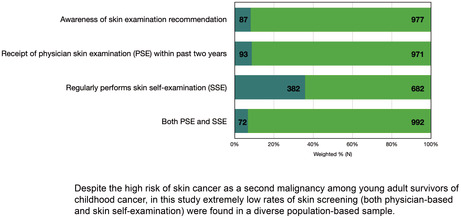

## 1 BACKGROUND

It is estimated that there are more than 500,000 childhood cancer survivors (CCS) living in the United States.^1^ Significant developments in the diagnosis and treatment of children with cancer have increased the 5‐year survival rate of pediatric cancer to over 80% in the past few decades.^2^, ^3^ Improved survival has been achieved through effective, yet complex and often toxic treatment regimens, causing significant complications and late effects of treatment such as heart failure, lung scarring, infertility, and second malignant neoplasms.^4^, ^5^

New malignancies are the most frequent cause of late mortality in patients who survived for more than 20 years after their childhood cancer diagnosis.^6^, ^7^, ^8^ Of secondary malignancies, skin cancer, including melanoma, basal cell carcinoma (BCC), and squamous cell carcinoma, is the most common.^9^ CCS have an approximately 83% increased risk of melanoma compared with the general population.^10^, ^11^ Further, CCS who were exposed to radiation have a nearly 30‐fold risk of developing skin cancer, primarily basal cell carcinomas (BCCs), with 46.7% of those with an initial BCC developing subsequent BCCs.^12^, ^13^ Morbidity and mortality outcomes among YA‐CCS who develop melanoma as a second malignancy depend upon tumor thickness (Breslow depth) and invasiveness of the tumor (as in general populations).^10^

Due to the high prevalence of skin cancer as a secondary malignancy for CCS, both the Children's Oncology Group (COG) and the National Cancer Institute (NCI) have issued guidelines for early prevention and detection of skin cancer for this at‐risk population. Guidelines include annual dermatological screening (physician‐based skin examination, or PSE), monthly self‐skin examination (SSE), as well as primary prevention of skin cancer (involving behaviors such as use of sunscreen and sun‐protective clothing to prevent excessive exposure to ultraviolet radiation).^14^, ^15^ In the few studies to date that have examined the prevalence of adherence to skin screening in this population, adherence to PSE has ranged from 11 to 29%, and from 16 to 48% for SSE.^6^, ^16^, ^17^, ^18^ As such, scientific and medical communities have called for increased awareness of and education among both CCS and their physicians to increase early detection for this high‐risk population.^19^, ^20^

There have been no population‐based studies examining adherence to recommended screening and surveillance for skin cancer among young adult childhood cancer survivors (YA‐CCS: diagnosed age 0–19 and currently age 18 or older^21^). Past studies have used clinical samples or have focused more broadly on multiple surveillance and sun protection behaviors.^6^, ^18^ Moreover, such studies have been set among predominantly non‐Latino white samples, despite significantly lower melanoma survival rates among people of color compared with non‐Latino whites.^22^ Thus, data regarding YA‐CCS of color and skin screening practices are lacking.^23^

We previously described adherence to and correlates of skin examination among an ethnically diverse population‐based sample of YA‐CCS melanoma survivors for whom both PSE and SSE constitutes primary surveillance.^24^ More than 80% of survivors practiced SSE, but only 65% reported recent PSE. In the present study, we focused on young adult survivors of cancer other than melanoma to identify the proportion who were aware of the need for skin cancer screening and to examine the prevalence and correlates of PSE and SSE, stratifying by receipt of radiotherapy because these survivors are at increased risk for skin cancer.^25^

## 2 METHODS

### 2.1 Data source

Data were obtained from Project Forward, a cross‐sectional population‐based cohort study examining engagement in cancer‐related follow‐up care and various psychosocial outcomes among YA‐CCS. Cases were identified through the Los Angeles Cancer Surveillance Program (LA CSP), the Surveillance, Epidemiology, and End Results (SEER) Cancer Registry covering LA County. Eligibility included patients diagnosed between the ages of 0 and 19 in LA County with any type of cancer, stage 2 or greater (except for brain cancer which was stage 1 or greater). Participants were mailed a survey available in both English and Spanish and received a $20 incentive to complete a survey. Participants were also entered into a lottery with a chance to win $300. Full study procedures have been previously described.^26^

For the present study, melanoma cases (*n* = 128) were excluded as routine skin examination already constitutes primary disease surveillance and were examined in a prior study.^24^ This study was approved by the California Committee for the Protection of Human Subjects of the California Cancer Registry and the Institutional Review Board at the University of Southern California.

### 2.2 Measures

#### 2.2.1 Outcomes: Awareness and receipt of PSE and SSE

To assess awareness of the need for skin examination, participants were asked about 7 COG‐recommended surveillance screening tests (“Are any of the following medical tests recommended for you in the future?”) including skin examination. To assess PSE, participants were provided with the same list of surveillance tests and asked, “in the past two years, did you receive any of the following medical tests?” Participants were asked to check all that apply, with “a skin exam (e.g., screening for skin cancer or mole check)” as one of the choices.

To assess SSE, participants were asked, “do you regularly check your skin for unusual moles, freckles, or spots?” Response options included “yes,” “no,” and “not sure.”

#### 2.2.2 Independent variables

Age and sex were obtained from cancer registry data, while race and ethnicity were self‐reported by study participants. Quintiles of socioeconomic status (SES; 1 = Lowest SES, 5 = Highest SES) were estimated using an established area‐based composite index comprising multiple socioeconomic indicators from census sources as described in previous literature.^27^, ^28^, ^29^ Insurance status was self‐reported and coded as insured versus uninsured.

Additional variables obtained from cancer registry data included date of diagnosis and stage of diagnosis. Stage of diagnosis was characterized using SEER summary staging (localized, regional and advanced stage) as the majority of cases were diagnosed prior to 2004 before American Joint Committee on Cancer (AJCC) staging was available in SEER data. Receipt of radiotherapy was obtained by self‐report and further validated from the cancer registry. In the few cases where differences arose, patient self‐report was used as registry data only includes the first 6 months of treatment received. Years since diagnosis was calculated by subtracting age at diagnosis from age at time of survey.

#### 2.2.3 Treatment intensity and late effects of treatment

Prior cancer treatment was categorized by the four‐level Intensity of Treatment Rating Scale 3.0 (ITR‐3; 1 = least intensive to 4 = most intensive) using a combination of self‐report and cancer registry data.^30^ Study participants were also provided with a list of common late effects of treatment (e.g., cardiotoxicities, auditory issues, reproductive issues, second malignancies) and were asked to note which they had experienced.^31^ The number of self‐reported late effects were summed into a scale ranging from 0 to 11.

#### 2.2.4 Healthcare utilization and engagement

Participants were asked whether they had received cancer‐related follow‐up care in the past 2 years; whether they had a regular source of non‐cancer care (e.g., a primary care provider); whether they discussed the health care they will need in the future with regards to the cancer treatments they received with their doctor; and whether they received a written treatment summary of their cancer therapy (all variables dichotomized as yes/no). All items were adapted from CCSS and have been validated in prior studies.^26^

Health care self‐efficacy (HCSE) was adapted from the Stanford Patient Education Research Center Chronic Disease Self‐Efficacy scales and consisted of three items related to survivors' perceived confidence in navigating the health care system.^32^ Responses comprised a 3‐point Likert‐type scale ranging from “not at all confident” to “totally confident.” Items were summed to form a continuous composite score ranging from 3 to 9 with higher scores indicating greater HCSE.

#### 2.2.5 Overall health

General health status was measured with a global item from the Short Form (36) Health Survey (SF‐36) asking participants to rate their overall health with a 5‐point Likert‐type scale ranging from “poor” to “excellent.”^33^

#### 2.2.6 Statistical Analysis

All analyses used sample weights to correct for non‐response bias (correcting for differences in the distribution of sex, race and ethnicity, and SES observed between responders and non‐responders).^26^ Descriptive frequencies and means were used to describe characteristics of the sample and prevalence of skin awareness and examination outcomes. Bivariate logistic regression models were conducted with variables selected for their potential significance to the outcomes; *p* < 0.10 was the entry threshold for the multivariable model.^34^ All models were adjusted for age at completion of survey, sex, SES, ethnicity and insurance status as covariates. All tests were two‐tailed, with an alpha less than 0.05 considered as significant. Analyses were conducted using SAS statistical software Version 9.4 (SAS Institute).

## 3 RESULTS

**FIGURE 1:**
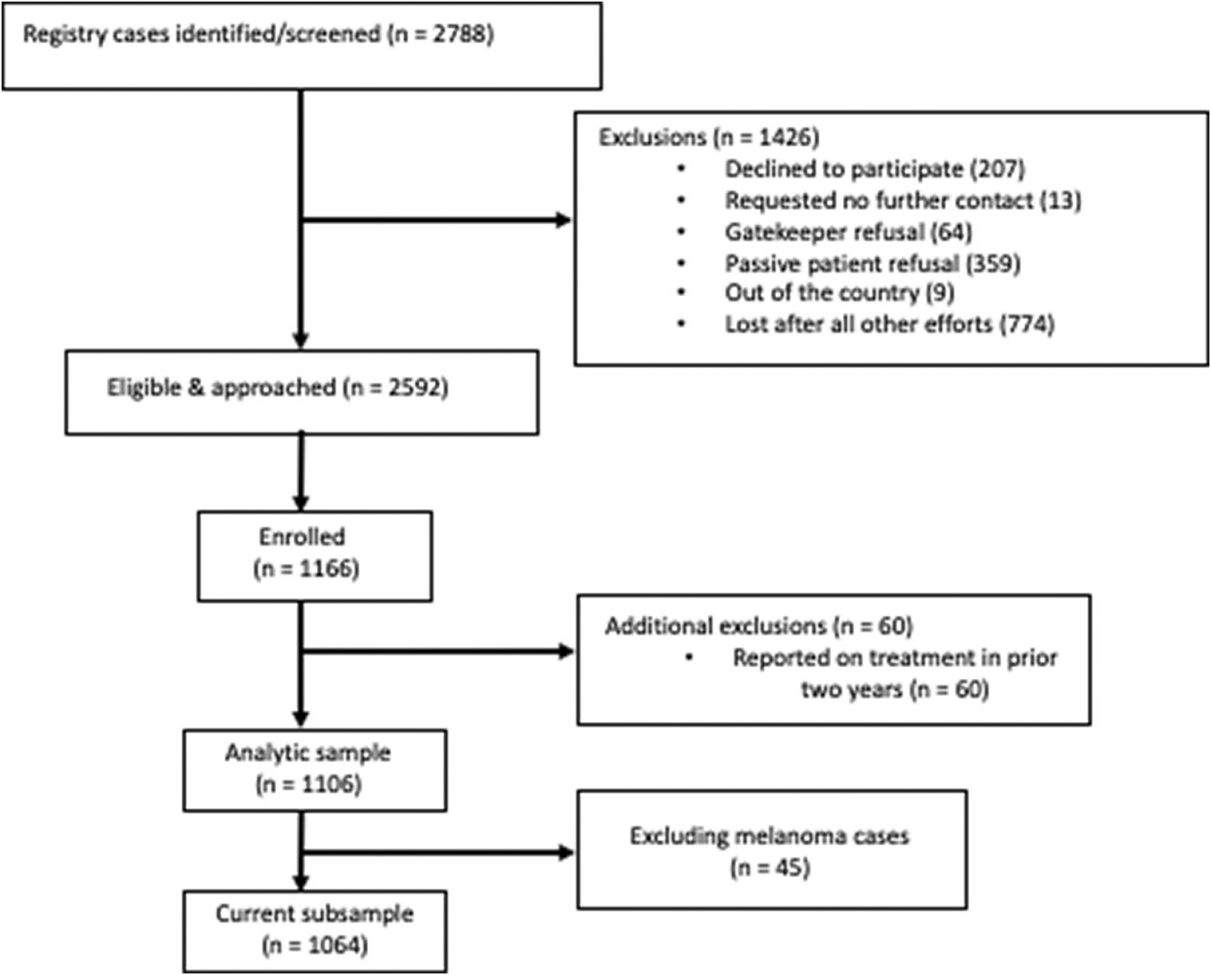
Recruitment flow chart.

Figure 1 shows the recruitment flow chart for the full sample and the current subsample (excluding melanoma patients). For the full sample, overall response rate was 44.9%, in line with other population‐based samples in this population.^35^ There were no differences between nonresponders and responders in age at diagnosis, years since diagnosis, age, cancer diagnosis, or stage of disease. Those who responded were more likely to be female, non‐Hispanic White, and have higher SES.^26^

Table 1 provides characteristics of the analytical subsample (*N* = 1064; excluding melanoma patients). Mean age at time of questionnaire completion was 25 years (SD ± 4.84) and mean time from diagnosis was 15 years (SD ± 4.37). Females comprised 51% of the sample and 27% of participants were non‐Latino white, 53% were Latino, and 20% were coded as other (including smaller numbers of Asian Americans, African Americans, Native Hawaiian or Pacific Islanders, American Indian or Alaska Native, or those identifying as multiracial). Twenty‐eight percent of the sample reported receiving radiotherapy (*N* = 293).

**TABLE 1:** Characteristics of sample (*N* = 1064)

	*N* (%)
Age at survey completion (M[SD])	25.06 (4.8)
Sex	
Female	542 (50.9)
Male	522 (49.1)
Socioeconomic status	
1st quintile (lowest)	339 (31.9)
2nd quintile	234 (22.0)
3rd quintile	160 (15.1)
4th quintile	170 (15.9)
5th quintile (highest)	161 (15.1)
Ethnicity	
White, non‐Latino	290 (27.3)
Latino	565 (53.1)
Other^a^	209 (19.6)
Cancer type	
Leukemia	392 (36.1)
Lymphoma	240 (21.7)
Brain and other nervous system	169 (15.2)
Endocrine system	60 (5.1)
Bones and joints	56 (5.0)
Genital system	56 (5.0)
Other^b^	91 (8.2)
Stage at diagnosis	
Local	146 (13.7)
Regional	339 (31.9)
Remote^c^	579 (54.4)
Years since diagnosis (M[SD])	14.56 (4.4)
Age at diagnosis (M[SD])	11.49 (5.4)
Treatment Intensity (ITR‐3)	
Level 1	33 (3.1)
Level 2	344 (32.3)
Level 3	539 (50.7)
Level 4	148 (13.9)
Received radiotherapy	
Yes	293 (27.5)
No	771 (72.5)
Late effects of treatment (M[SD])	0.76 (1.3)
Follow‐up care in past 2 years	
Yes	612 (58.5)
No	434 (41.5)
Regular source of non‐cancer care	
Yes	756 (72.3)
No	290 (27.7)
Discuss future healthcare with doctor	
Yes	540 (51.7)
No/Don't Know	505 (48.3)
Provided a written treatment summary	
Yes	474 (45.4)
No/Don't Know	571 (54.6)
Health care self‐efficacy (M[SD])	4.84 (1.4)
Self‐rated health (M[SD])	2.33 (1.0)

^a^ Includes African American, American Indian, Middle Eastern, Pacific Islander, and other/unknown.

^b^ Oral cavity and pharynx, digestive system, respiratory system, soft tissue including heart, urinary system, eye and orbit and miscellaneous.

^c^ Includes high‐risk leukemias.

### 3.1 Prevalence of skin examination awareness and receipt

Eight percent of participants were aware of the need for skin examination, 9% reported receipt of PSE within the past 2 years, and 35% reported performing SSE regularly (Table 2). Six percent reported engagement in both PSE and SSE.

**TABLE 2:** Skin examination in young adult survivors of childhood cancer (*N* = 1064)

	*N* (Weighted %)
Awareness of skin examination recommendation	
Yes	87 (8.0)
No	977 (92.0)
Receipt of physician skin examination (PSE) within past 2 years	
Yes	93 (8.5)
No	971 (91.5)
Regularly performs skin self‐examination (SSE)	
Yes	382 (35.4)
No/not sure	682 (64.6)
Both PSE and SSE	
Yes	72 (6.4)
No/not sure	992 (93.6)
Among YACCS exposed to radiotherapy (*N* = 293)	
Awareness of skin examination recommendation	
Yes	31 (9.9)
No	262 (90.1)
Receipt of physician skin examination (PSE) within past 2 years	
Yes	31 (10.2)
No	262 (89.8)
Regularly performs skin self‐examination (SSE)	
Yes	113 (37.9)
No/not sure	180 (62.1)
Both PSE and SSE	
Yes	24 (7.6)
No/not sure	269 (92.4)

Among YA‐CCS who received radiotherapy, 10% reported awareness of the need for skin examination, 10% reported receipt of PSE, and 38% reported regular SSE. Eight percent reported engagement in both PSE and SSE.

### 3.2 Regression models

Table 3 presents the bivariate and multivariable regression models with PSE, SSE, and both PSE and SSE as outcomes. In multivariable analyses with PSE as the outcome, higher SES, greater treatment intensity, greater number of late effects, recent cancer follow‐up visit, receipt of a written treatment summary, and greater HCSE were significantly positively associated with PSE. Latino ethnicity was significantly negatively associated with PSE. In multivariable analyses with SSE as the outcome, greater number of late effects, receipt of a written treatment summary, and greater HCSE were significantly associated with SSE.

**TABLE 3:** Bivariate and multivariable models of correlates of skin examination among young adult survivors of childhood cancer (*N* = 1064)

	Physician skin exam (PSE)		Self‐skin exam (SSE)		Adherence to PSE & SSE	
	Bivariate	Multivariable	Bivariate	Multivariable	Bivariate	Multivariable
	OR [95% CI]	AOR [95% CI]	OR [95% CI]	AOR [95% CI]	OR [95% CI]	AOR [95% CI]
Demographic characteristics						
Current age at time of survey^a^	1.02 (0.95–1.08)	1.02 (0.96–1.08)	0.98 (0.97–1.01)	0.99 (0.97–1.01)	1.03 (0.95–1.11)	1.03 (0.96–1.11)
Sex (ref, male)^b^	1.07 (0.74–1.56)	1.01 (0.71–1.43)	1.23 (1.01–1.50)*	1.20 (0.96–1.50)	1.22 (0.85–1.75)	1.17 (0.85–1.60)
Socioeconomic status (lowest to highest quintile)^c^	1.23 (1.05–1.44)*	1.25 (1.04–1.50)*	1.08 (0.99–1.19)+	1.03 (0.92–1.16)	1.24 (1.03–1.50)*	1.22 (1.00–1.48)*
Latino (ref, non‐Latino)^b^	0.62 (0.44–0.87)**	0.58 (0.41–0.81)**	0.82 (0.61–1.11)	0.75 (0.54–1.05)+	0.58 (0.37–0.90)*	0.56 (0.36–0.86)**
Insured (yes/no)^b^	1.69 (0.74–3.86)	1.07 (0.50–2.27)	1.36 (0.95–1.94)+	1.02 (0.70–1.49)	2.72 (1.12–6.59)*	1.60 (0.74–3.45)
Clinical characteristics						
Stage at diagnosis (local to distant)^c^	1.16 (0.91–1.48)	—	1.10 (0.96–1.26)	—	1.16 (0.79–1.70)	—
Years since diagnosis^a^	0.98 (0.89–1.07)	—	1.00 (0.94–1.05)	—	0.98 (0.87–1.10)	—
Age at diagnosis^a^	1.03 (0.93–1.12)	—	1.00 (0.95–1.06)	—	1.02 (0.91–1.15)	—
Treatment intensity (lowest to highest)^c^	2.05 (1.54–2.74)***	1.81 (1.36–2.40)***	1.21 (0.94–1.57)	—	1.92 (1.34–2.76)***	1.70 (1.21–2.40)**
Received radiotherapy (y/n)^b^	1.28 (0.87–1.89)	—	1.20 (0.88–1.63)	—	1.22 (0.83–1.78)	—
Late effects of treatment (0‐11)^a^	1.39 (1.28–1.51)***	1.34 (1.19–1.50)***	1.15 (1.05–1.26)**	1.21 (1.09–1.34)***	1.32 (1.18–1.49)***	1.29 (1.14–1.47)***
Healthcare characteristics						
Cancer follow‐up care in past 2 years (y/n)^b^	2.49 (1.75–3.53) ***	1.79 (1.25–2.57)**	1.65 (1.38–1.98)***	1.23 (0.98–1.64)+	2.60 (1.63–4.15)***	1.66 (1.07–2.55)*
Regular source of non‐cancer care (y/n)^b^	0.90 (0.47–1.72)	—	1.26 (0.94–1.70)	—	0.82 (0.33–2.04)	—
Discuss future health care with physician (y/n)^b^	1.44 (0.82–2.52)	—	1.44 (1.22–1.69)***	1.10 (0.88–1.36)	2.30 (1.38–3.83)**	1.56 (0.93–2.62)+
Written treatment summary (y/n)^b^	1.86 (1.34–2.59) ***	1.48 (1.02–2.14)*	1.76 (1.44–2.16)***	1.46 (1.19–1.80)***	2.03 (1.37–3.01)***	1.38 (0.97–1.96)+
Health care self‐efficacy (low to high)^a^	1.30 (1.14–1.48)***	1.27 (1.09–1.48)**	1.36 (1.25–1.48)***	1.28 (1.15–1.41)***	1.60 (1.34–1.92)***	1.56 (1.24–1.97)***
Self‐rated health (poor to excellent)^c^	0.93 (0.79–1.11)	—	1.17 (1.03–1.32)**	1.12 (0.98–1.28)+	1.07 (0.81–1.43)	—

*Note*: Sample weights were applied. All models adjusted for age at time of survey, sex, SES, ethnicity, insurance status, and clustering at diagnosing hospital.

^a^ Continuous variable.

^b^ Dichotmous variable.

^c^ Ordinal variable.

*+p* < 0.10; *p < 0.05, **p < 0.01, ***p < 0.001.

For both PSE and SSE, in multivariable analyses, higher SES, greater treatment intensity, greater number of late effects, recent cancer‐related follow‐up visit, receipt of a written treatment summary, and greater HCSE were significantly positively associated with both exams. Latino ethnicity was significantly negatively associated with both exams.

## 4 DISCUSSION

The present study assessed the prevalence and correlates of awareness of and adherence to skin cancer surveillance among a population‐based sample of ethnically diverse YA‐CCS. Overall, skin cancer screening rates were low, with only 8% reporting awareness of the need for screening, 9% and 35% reporting PSE and SSE, respectively, and 6% reporting adherence to both PSE and SSE. Awareness and adherence were not substantially higher (at 10%, 10% and 38%) among YA‐CCS who had received radiotherapy, the highest risk subgroup for developing skin cancer (among this subgroup which excluded melanoma cases).

While estimates of PSE and SSE in the general population vary, the prevalence of ever having either PSE or SSE ranges from 14 to 21% in average risk adults.^36^, ^37^, ^38^ Among childhood cancer survivors, the prevalence of skin cancer screening in this study was lower than in four prior studies conducted among clinically recruited samples (with three using data from the Childhood Cancer Survivor Study [CCSS] and one recruiting from a long‐term follow‐up program at an academic medical center). Buchanan et al. found that 29% of YA‐CCS were ever examined for skin cancer.^17^ Nathan et al. found that 27% of high‐risk YA‐CCS (those who had received radiation therapy) reported PSE.^6^ Among a radiation‐treated sample of 728 YA‐CCS, Geller et al. found that 16.4% and 11.0% reported SSE or a PSE, respectively, in the prior 12 months, and 13% reported having both examinations in the prior 12 months.^16^ Stapleton et al. found that 31% of YA‐CCS had ever received a PSE and approximately 48% had ever conducted a SSE.^18^ The present study differed in that it was population‐based (recruited from SEER), included all cancer types (as opposed to a selected group of cancer diagnoses as in the CCSS), and included those patients not currently engaged in healthcare.^39^ Further, prior studies had limited representation of ethnic and racial minoritized patients, with representation ranging from 8 to 20% participants of color, whereas our study represented a diverse population, with a sample greater than 50% Latino. Thus, our figures represent an estimate that may be closer to the prevalence of skin screening when including those who have dropped out of cancer surveillance and among non‐white YA‐CCS.

In adjusted models, several healthcare factors were associated with both adherence to PSE and SSE. Greater healthcare self‐efficacy was associated with the likelihood of recent screening, suggesting that YA‐CCS who had more confidence to navigate the healthcare system were more likely to perform both modes of skin surveillance, while those who had received a written treatment summary were significantly more likely to perform SSE. YA‐CCS with greater treatment intensity were more likely to receive PSE, and those who reported a greater number of late effects from treatment were more likely to engage in both PSE and SSE. Collectively, these findings suggest that YA‐CCS who are enfranchised within the healthcare system either through self‐management, survivorship informational resources, or due to ongoing health needs, and from higher risk groups, may be more likely to receive counseling on and subsequently engage in recommended skin care screening. As YA‐CCS drop out of cancer‐focused survivorship care at high rates as age and time from diagnosis increases,^26^, ^40^, ^41^ our findings underscore the need to retain this population in healthcare to receive needed preventive care such as skin examination.

Whereas prior research among this sample found that Latino YA‐CCS were 31% less likely to engage in recent cancer‐focused survivorship care than non‐Latino white YA‐CCS,^26^ in this study Latino YA‐CCS were 45% less likely to report receiving PSE than non‐Latino whites, controlling for SES. Latino YA‐CCS experience greater health disparities and barriers in access to care than their non‐Latino white counterparts,^26^, ^42^, ^43^, ^44^ and non‐cancer affected Latinos exhibit lower risk awareness and skin cancer protection behaviors than non‐Latino white populations.^45^, ^46^, ^47^, ^48^ Thus, Latino YA‐CCS and potentially those from other ethnic or racially minoritized populations may be at increased risk for later detection of skin cancer leading to invasive disease and poorer outcomes.

In alignment with other studies, we found higher rates of SSE than PSE. While PSE is the gold standard for skin examination, given the barriers identified in the literature to PSE, SSE may be a more amenable or practical target for behavior change in YA‐CCS to enhance early detection. SSE has been associated with earlier stage of diagnosis and reduced mortality from melanoma and may be an efficient screening practice for this population who experience multiple healthcare barriers to follow‐up care after cancer including cost, lack of information (such as receipt of a survivorship care plan), insurance instability/coverage, and issues with transition from pediatric to adult care settings.^26^, ^40^, ^49^, ^50^, ^51^ Expanding patient counseling regarding frequent, whole‐body SSE (with training in performance of comprehensive skin examination and visual aids of suspicious lesions), and targeting those YA‐CCS at highest risk of developing skin cancer may prove to be an important and feasible way to improve early detection for these high‐risk patients while continuing to educate and raise awareness of the need for annual PSE.

Strengths of the study include a large, cancer‐registry derived, and ethnically diverse population‐based sample weighted to address non‐response bias. Study limitations include the cross‐sectional design which does not allow causal inference. Additionally, the lack of objective data (e.g., medical chart abstraction) was a limitation of the study. Generalizability of findings may be limited to regions similar to LA County, i.e., other large urban areas in the U.S. with high proportions of Latino populations. Our measures for both PSE and SSE assessed only recent engagement in these behaviors and did not provide a qualitative appraisal of their ability to detect skin cancer. Future research should examine whether YA‐CCS who engage in PSE and SSE are meeting dermatologic and oncologic recommendations for total body skin examination such that these measures would effectively detect skin cancer at an early stage

In conclusion, among a population‐based sample of diverse YA‐CCS, adherence to recommended screening for skin cancer was extremely low. Greater awareness and the development of effective strategies are needed to greatly expand screening in this at‐risk population to ensure that this common and serious secondary malignancy is detected at an earlier and curable stage.

## AUTHOR CONTRIBUTIONS

**Angela A Li:** Formal analysis (supporting); writing – original draft (supporting); writing – review and editing (supporting). **Katherine Wojcik:** Data curation (lead); writing – review and editing (supporting). **Julia Stal:** Writing – original draft (supporting); writing – review and editing (supporting). **Myles Cockburn:** Writing – review and editing (supporting). **Gino K. In:** Writing – review and editing (supporting). **David R Freyer:** Writing – review and editing (supporting). **Ann S. Hamilton:** Writing – review and editing (supporting). **Joel Milam:** Funding acquisition (lead); writing – review and editing (supporting).

## FUNDING INFORMATION

This paper was supported by grant 1R01MD007801 from the National Institute on Minority Health and Health Disparities of the National Institutes of Health. Additional support was provided by P30CA014089 from the National Cancer Institute of the National Institutes of Health. K.Y.W. was supported by training Grants T32CA092408 and T32CA009492 from the National Cancer Institute of the National Institutes of Health.

## CONFLICT OF INTEREST

The authors declare that they have no conflict of interest.

## Data Availability

Data sharing will be considered by the PI with reasonable request and with the approval of the California Cancer Registry.
